# HIV Care in Ukrainian Migrants in Two European Countries: All the Same?

**DOI:** 10.3390/pathogens13080621

**Published:** 2024-07-26

**Authors:** Kathrin van Bremen, Miłosz Parczewski, Malte Monin, Magdalena Leszczyszyn-Pynka, Stefan Schlabe, Franciszek Lenkiewicz, Malwina Karasińska-Cieślak, Jan-Christian Wasmuth, Magdalena Witak-Jędra, Sven Breitschwerdt, Jürgen K. Rockstroh, Dmytro Zhyvytsia, Christoph Boesecke, Daniel Chober, Bogusz Aksak-Wąs

**Affiliations:** 1 Bonn University Hospital, 53127 Bonn, Germany; kathrin.vanbremen@ukbonn.de (K.v.B.); malte_benedikt.monin@ukbonn.de (M.M.); stefan.schlabe@ukbonn.de (S.S.); jan-christian.wasmuth@ukbonn.de (J.-C.W.); sven.breitschwerdt@ukbonn.de (S.B.); juergen.rockstroh@ukbonn.de (J.K.R.); christoph.boesecke@ukbonn.de (C.B.); 2 German Centre for Infection Research (DZIF), 53127 Bonn, Germany; 3 Department of Infectious, Tropical Diseases and Acquired Immunodeficiency, Pomeranian Medical University in Szczecin, 71-455 Szczecin, Poland; milosz.parczewski@pum.edu.pl (M.P.); malwina_k_cieslak@wp.pl (M.K.-C.); kalendrial@gmail.com (D.C.); 4 Department of Infectious, Tropical Diseases and Immune Deficiency, Provincial Hospital, 71-455 Szczecin, Poland; mlpynka@interia.pl (M.L.-P.); magdalenka72@o2.pl (M.W.-J.); dzhyvytsia@gmail.com (D.Z.)

**Keywords:** HIV, migration, Germany, Poland, Ukraine, war

## Abstract

**Introduction:** War in Ukraine prompted an enormous refugee influx into Europe, including approximately 4200 people with HIV. The unique healthcare features of Ukrainian refugees living with HIV were compared between two infectious disease departments in Bonn, Germany, and Szczecin, Poland. **Methods:** This is a retrospective study on 161 people living with HIV (PLWH) refugees from Ukraine seeking care in Bonn (n = 30) and Szczecin (n = 131) between April 2022 and May 2023. Demographic, virologic, immunologic, and coinfection data were analyzed. **Results:** The majority of the studied individuals were female: 64% (n = 84) in Szczecin and 60% (n = 18) in Bonn. The main HIV transmission mode was heterosexual sex in 73.5% (n = 114). All were on combined antiretroviral therapy (cART) on arrival, primarily on the TLD regimen (TDF/3TC/DTG) (68.4%, n = 106). In Germany, cART was most frequently switched to BIC/TAF/FTC in 83.4% (n = 25); in Poland, the most common combination was TDF/FTC + DTG (58%, n = 76). A prevalence of replicating hepatitis C was in 11.7% (n = 15), and that for chronic hepatitis B (HBV) was in 4.7% (n = 4). History of past tuberculosis was reported in 16.9% (n = 14, Poland, and n = 7, Germany). Follow-up after 6 months showed immunological reconstitution with a mean increase of CD4+ of 10 (IQR: −69.5–120.5) cells/µL in Poland and 51.5 (IQR: −22.5–135.5) cells/µL in Germany; *p* = 0.04. Virologic suppression (<40 HIV-RNA/mL) was high in care entry (n = 62; 98%) for Poland, and n = 26 (92.6%) for Germany, and suppression was achieved in the majority of patients in the 6-month control (89.7% in Poland vs. 95.7% in Germany). **Conclusions:** Health challenges posed by war migration extend beyond HIV to coinfections as HBV, HCV, and tuberculosis give an indication for a broader search for coinfections, often less common in the new country.

## 1. Introduction

The ongoing war in Ukraine has had myriad ramifications on the global stage. Among the most palpable consequences has been the massive movement of refugees seeking asylum across different European nations [1]. Migration of such a vast population inherently entails various public health challenges. One such crucial concern arises from the movement of people with HIV (PLWH) and the subsequent implications for both healthcare systems and the individuals involved [1,2]. The number of people living with HIV in Ukraine is over 250,000 [3] compared to Poland [4], where the number is just over 25,000, and Germany, where the number is just over 90,000 [5]. The number of HIV-infected patients currently living in Ukraine is difficult to estimate due to the destruction of a large part of the medical infrastructure; these figures take into account statistics before the outbreak of the war. In Ukraine, care for patients living with HIV was widely spread, not limited to large cities like, for example, in Poland. Even more so, the war destroyed a very large percentage of the available medical infrastructure and forced patients to move, often losing their physician and medical documentations.

The continuity of care for PLWH is crucial, given the implications of treatment interruptions on both individual health and public health grounds. Interruptions can lead to viral rebound, progression to AIDS with resulting death, as well as the emergence of drug-resistant strains [6].

Human immunodeficiency virus (HIV) care encompasses more than just antiretroviral therapy. Regular monitoring of virologic and immunologic indices, addressing coinfections like hepatitis B (HBV) and hepatitis C (HCV), and managing associated comorbidities constitute integral facets of comprehensive HIV care [7,8]. 

Thus, despite well-established service in HIV care in Germany and Poland, the integration of refugees with HIV presented unique challenges. While both nations adhere to the overarching guidelines stipulated by international bodies like the World Health Organization or European AIDS Clinical Society (EACS) [9], the nuances in clinical practices, healthcare infrastructure, drug availability, and specific treatment protocols can vary. Moreover, language barriers complicate HIV care.

Furthermore, the psychosocial dimensions associated with being a refugee—trauma, displacement, cultural differences, language barriers, and potential stigma associated with being HIV-positive—can profoundly impact treatment adherence, medical follow-ups, and overall health outcomes.

Access to healthcare in both countries is universal; both entities provide care as part of free healthcare. War migrants are covered by health insurance, ARV treatment is free, and its implementation has not been postponed in any of the centers.

This study seeks to elucidate the healthcare features and outcome of Ukrainian PLWH refugees in two representative infectious disease departments in Bonn, Germany, and Szczecin, Poland. The analysis aims to shed light on demographic profiles, predominant modes of HIV transmission, initial and subsequent ARV regimens, and the burden of associated coinfections.

## 2. Methodology

### 2.1. Study Design and Population

This retrospective cross-sectional study was carried out on a cohort of 161 HIV-positive Ukrainian refugees who sought medical care at two infectious disease departments in Bonn, Germany (n = 30), and Szczecin, Poland (n = 131), between April 2022 and May 2023. All people diagnosed with HIV in Ukraine who then moved to Szczecin, Poland, or Bonn, Germany, during the war were included in the analysis. No exclusion criteria were applied.

### 2.2. Data Collection and Parameters

Patient-level data were systematically collected, encompassing demographics, mode of HIV transmission, virological and immunological metrics, hepatitis coinfection status, and prior tuberculosis history. Moreover, their current treatment regimens were recorded. CD4+ cell counts were measured via flow cytometry; HIV-RNA was measured employing local assays (Abbott in Bonn; Abbott Molecular (Des Plaines, IL, USA); Gene X-pert or Cobas Roche Diagnostics in Szczecin Roche Molecular Systems, Inc. (Mannheim, Germany)).

### 2.3. Statistical Analysis

Descriptive statistics were used for the summative analysis of the sample and observations. Continuous variables, such as age and CD4+ cell counts, were expressed as median with interquartile range (IQR), while categorical data, such as gender and mode of HIV transmission, were presented as mean values and percentages. Statistical analyses were performed for nominal variables using a chi-square test (sex, transmission route, ARV used, and presence of coinfections), while continuous variables (age and CD4+ lymphocyte count) were analyzed using a Mann–Whitney U-test (Statistica v12 software; Statsoft, Tulsa, OK, USA). Statistical significance was set at *p* < 0.05.

This study was conducted according to the Declaration of Helsinki. 

## 3. Results

### 3.1. Baseline Characteristics

Baseline characteristics are found in Table 1. The median age of the individuals was 41 years (IQR: 35–47) in Szczecin and 40.5 years (IQR: 38–46) in Bonn, with no statistically significant difference between the two groups (*p* = 0.99). Concerning gender, females constituted 64% in Szczecin and 60% in Bonn, with no significant difference observed (*p* = 0.67).

### 3.2. Transmission and Treatment

Heterosexual transmission was the most prevalent mode of HIV acquisition, accounting for 79% (n = 102) in Szczecin and 50% (n = 14) in Bonn. Intravenous drug abuse (IVDA) was seen in 12.4% (n = 16) of the patients in Szczecin vs. 39.2% (n = 11) in Bonn. Transmission through homosexual sex between men (MSM) was responsible for 8.6% (n = 11) and 7.2% (n = 2) of HIV infections, respectively. Mother-to-child transmission was noted only in one case in Bonn. Statistical significance was seen throughout the distribution of mode of HIV acquisition between those two centers with *p* = 0.002.

Upon arrival, all refugees were on combined antiretroviral therapy (cART), with the TDF/3TC/DTG regimen being most common in both centers (72.4%; (n = 94) in Szczecin; 85.2% (n = 23) in Bonn (*p* = 0.004)).

### 3.3. Virologic and Immunologic Parameters upon First Presentation

At arrival, the median CD4+ count was 566 cells/µL (IQR: 358–751) in Szczecin and 492.5 cells/µL (IQR: 367–670) in Bonn, with no statistically significant difference (*p* = 0.42). Viral suppression, defined as <40 HIV-RNA/mL, was achieved in 98.4% (n = 62) of individuals in Szczecin and 92.6% (n = 26) in Bonn.

### 3.4. Coinfections

Active HCV coinfection was present in 11.7% (n = 12) of the individuals in Szczecin and 16.7% (n = 5) in Bonn (*p* = 0.23), tested with a screening method for anti-HCV antibodies, if positive molecular testing of HCV RNA was performed. HBV coinfection was observed in 4.2% (n = 3) of the individuals in Szczecin and 3.3% (n = 1) in Bonn, screened with hepatitis B surface antigen (HBsAg), hepatitis B surface antibody (anti-HBs), and hepatitis B core antibody (anti-HBc). Additionally, past tuberculosis was self-reported in 14.1% (n = 14) of the patients in Szczecin and 27.6% (n = 8) in Bonn, with no significant difference between the two centers (*p* = 0.09). As routine procedure, all patients were screened as stated.

### 3.5. Changes over Time (6–12 Months in EU)

At 6–12 months post-arrival (Table 2), immunological reconstitution was observed in 51.1% (n = 45) of the patients in Szczecin and 62.9% (n = 17) in Bonn (*p* = 0.28). The mean increase in CD4+ cell counts was 10 cells/µL (IQR: −69.5–120.5) in Szczecin and 51.5 cells/µL (IQR: −22.5–135.5) in Bonn, without statistically significant differences between the two sites (*p* = 0.36). The proportion of patients with undetectable viremia (<50 copies/mL) after the 6-12-month observation was 89.8% (n = 79) in Szczecin and 95.7% (n = 22) in Bonn (*p* = 0.04). Nonetheless, 31% (n = 48) of patients were lost to follow-up. The lost to follow-up rate in Poland (32.8%; n = 43) was higher than that in Germany (20.8%; n = 5). 

## 4. Discussion

The interplay between geopolitical events and global health often finds itself at the nexus of public health challenges. The results from our study unravel several pivotal aspects related to the healthcare features of Ukrainian refugees with HIV in Bonn, Germany, and Szczecin, Poland.

One striking observation is the prominence of heterosexual transmission as the primary mode of HIV transmission, especially among the cohort in Szczecin. This observation underscores the evolving epidemiology of HIV where previously certain modes of transmission, such as MSM (men who have sex with men) or IDU, may have dominated specific demographics [1]. The greater number of HIV-infected women is most likely to be interpreted in line with the higher rate of heterosexual transmission in Ukraine and also men not being allowed to leave Ukraine for combat reasons. Although the vast majority of war refugees were women, among men, heterosexual contact was also the dominant route of HIV transition. Thus, it underlines the fact of low-threshold screening of women from Ukraine to detect infection at earlier stages. Moreover, four women in Bonn were diagnosed newly after arrival and all presented late with CD4 cells <200/µL. The prominence of heterosexual transmission warrants tailored public health strategies targeting this demographic both in the context of prevention and management. Heterosexual transmission has been associated with late HIV diagnosis and therefore needs special attention as heterosexual persons might not consider themselves at risk due to their lower attention towards HIV transmission risks [7].

The uniformity in the usage of ARV regimens among the refugees upon arrival suggests a consolidated approach to HIV management in Ukraine. TLD (TDF/3TC/DTG) is also available at a lower cost in Ukraine, but unfortunately, this is not the case in Poland or Germany. The widespread use of the TLD regimen is consistent with global trends, given the regimen’s efficacy and reduced side effect profile [10]. However, the variation observed in the subsequent switch of ARV regimens between the two European nations is intriguing. It sheds light on the nuanced differences in clinical protocols and the drug availability and accessibility between the countries. It must be questioned which regime is best for PLWH from Ukraine who plan to return to their home in the future as BIC/FTC/TAF is not available in Ukraine. The other option is to switch to a two-tablet regimen consisting of TDF/FTC plus DTG while abroad. Moreover, caution should be taken in switching PLWH from Ukraine to a dual regimen with DTG/3TC, as the centers in Germany and Poland lack pre-treatment history as well as resistance testing. According to national guidelines, most patients in Germany have their ARV therapy changed within a single-tablet regimen (most often to BIC/FTC/TAF), while in Poland, it is suggested that the changes be as small as possible; so, most patients have the TLD regimen split, with a change from lamivudine to emtricitabine and further continuing with the two-tablet regimen, i.e., TDF/FTC and DTG.

The approach to testing war refugees in both countries is different: in Poland, there is a program for free and anonymous testing for HIV, HCV, and syphilis infection, but it is not directly addressed to refugees. In Germany, refugees are screened for tuberculosis and other coinfections, which may result in better access to healthcare.

The high prevalence of hepatitis coinfections, particularly HCV, underlines the complex web of challenges these refugees present. HCV—with its known mutual interference with HIV, leading to higher rates of liver damage and liver cirrhosis, with resulting complications such as portal hypertension and hepatocellular carcinoma (HCC)—demands vigilant screening. A large percentage of patients infected with HCV corresponds to a large number of patients infected through intravenous drug use. It has been postulated in recent years that up to one-quarter of people abusing intravenous drugs may be infected with HCV in Ukraine. As European guidelines recommend treatment of every HIV/HCV-coinfected patient, every country will have to consider how to solve those problems as costs of HCV treatment could lead to public health challenges. The high rate of HCV-coinfection accentuates the need for an integrated approach to care, where HIV management is harmoniously synchronized with the management of coinfections. Additionally, there has to be effort in bringing PLWH with ongoing drug use to opiate substitution to decrease the risk of HCV infection and transmission. Concerning HBV, vaccination rates should be increased as the rate of vaccination was very low in the study group (2.8% in Szczecin and 0% in Bonn). In comparison, the rate of successful HBV vaccination in the Bonn cohort is 64.3% [11].

Considering immunological aspects, the CD4+ counts observed in both cohorts upon arrival were commendably within a manageable range. This suggests that the refugees, despite the upheavals of war and migration, had relatively sustained healthcare access in Ukraine [12]. The subsequent immunological reconstitution during their European stay, although not markedly different between the two nations, denotes the effectiveness of the healthcare systems in assimilating and managing these refugees. Moreover, the high rate of sustained virologic control with HIV loads below the detection rate of 50 copies/mL underlines good adherence to PLWH from Ukraine. It can be stated that refugees from Ukraine remain with good adherence despite major challenges (e.g., language barrier, socio-economic problems, and stigma) brought up by war migration.

However, the number of patients lost to follow-up is a concern. It highlights the potential barriers to continuous care, which could range from socio-economic challenges, fear of stigma, cultural and language barriers to the transient nature of refugee status as well as ongoing IVDA resulting in compliance difficulties.

This study has some limitations. Firstly, it represents only a single center’s experience in each of the two countries, with comparably low numbers in Germany. Secondly, the rate of patients who were lost to follow-up is of concern. This might result from cultural and language barriers as well as the transient nature of refugee status. Thus, this study represents a real-life cohort of war refugees living with HIV and their special needs.

## 5. Conclusions

The healthcare features of Ukrainian PLWH refugees in Germany and Poland present a tableau of both commendable resilience and impending challenges. While the high rates of viral control and sustained immune function are heartening, the prevalence of coinfections like HBV, HCV, and tuberculosis underline the multifaceted nature of care required by these individuals. Additionally, special attention has to be paid towards low-threshold testing in women, who are at higher risk due to different transmission risks between the countries.

Differences in antiretroviral regimens between the countries reflect the diversity in clinical practices across Europe, even within a unified framework of global guidelines. However, the overarching theme remains: the need for an integrated, holistic approach to manage not just HIV but the spectrum of associated conditions and challenges.

## Figures and Tables

**Table 1:** Comparison of patients diagnosed in Ukraine in terms of viral and bacterial coinfections, immunological and virological status (tests performed in Poland and Germany) and implemented antiretroviral therapy.

Data	Szczecin, PL (n: 131)	Bonn, DE (n: 30)	*p* Value
Age (mean. IQR)	41 (IQR: 35–47)	40.5 (IQR: 38–46)	*p* = 0.99
Gender			
Female; %	84 (64%)	18 (60%)	*p* = 0.67
Male; %	47 (36%)	12 (40%)	
Mode of HIV transmition			
Hsx; %	102 (79.0%)	14 (50%)	*p* = 0.002
IVDA; %	16 (12.4%)	11 (39.2%)	
MSM; %	11 (8.6%)	2 (7.2%)	
MTCT; %	0 (0%)	1 (3.6%)	
Year of diagnosis (mean. IQR)	2014 (IQR: 2009–2018)	2010 (IQR: 2006–2017)	*p* = 0.02
Year of ARV start (mean. IQR)	2016 (IQR: 2011–2018)	2013 (IQR: 2010–2017)	*p* = 0.1
CD4+/µL upon arrival. (mean. IQR)	566 (IQR: 358–751)	492.5 (IQR: 367–670)	*p* = 0.42
ARV upon arrival			
DTG/3TC/TDF	94 (72.4%)	23 (85.2%)	*p* = 0.004
DTG/3TC	1 (0.7%)	0 (0%)	
ABC/3TC/DTG	7 (5.4%)	1 (3.7%)	
TDF/FTC/EFV	15 (11.5%)	0 (0%)	
LPV/r/TDF/FTC	0 (0%)	1 (3.7%)	
TDF/FTC/RPV	0 (0%)	2 (7.4%)	
TDF/FTC + DTG	11 (8.5%)	0 (0%)	
TDF/FTC + RAL	2 (1.5%)	0 (0%)	
ARV after change in EU			
TAF/FTC/BIC	26 (19.8%)	25 (83.4%)	*p* < 0.0001
DTG/3TC	7 (5.4%)	1 (3.3%)	
TDF/FTC/RPV	0 (0%)	1 (3.3%)	
ABC/3TC/DTG	5 (3.8%)	1 (3.3%)	
TDF/FTC + DTG	76 (58%)	0 (0%)	
TDF/FTC + RAL	2 (1.6%)	0 (0%)	
DTG/3TC/TDF	7 (5.4%)	2 (6.7%)	
TDF/FTC/EVG/c	1 (0.7%)	0 (0%)	
TDF/3TC/DOR	6 (4.6%)	0 (0%)	
TAF/FTC/RPV	1 (0.7%)	0 (0%)	
Undetectable VL upon arrival	62 (98.4%)	26 (92.6%)	*p* = 0.25
HCV infection status			
No HCV infection	67 (65%)	16 (53.3%)	*p* = 0.5
Active HCV	12 (11.7%)	5 (16.7%)	
Resolved HCV	24 (23.3%)	9 (30%)	
HBV infection status			
No HBV infection	50 (70.4%)	16 (53.4%)	*p* = 0.23
Active HBV	3 (4.2%)	1 (3.3%)	
OBI (only a/Hbc total positive) isolated anti-HBc	9 (12.7%)	9 (30%)	
OBI (a/Hbc tot positive + a/Hbs positive)/resolved HBV	7 (9.9%)	4 (13.3%)	
Vaccinated	2 (2.8%)	0 (0%)	
TBC status			
History of TBC	14 (14.1%)	8 (27.6%)	*p* = 0.09
No history of TBC	85 (85.9%)	21 (72.4%)	

PL: Poland; DE: Germany; EU: European Union; IQR: interquartile range; Hsx: heterosexual; IVDA: intravenous drug abuse; MSM: men who have sex with men; MTCT: mother-to-child transmission; ARV: antiretroviral treatment; DTG: dolutegravir; 3TC: lamivudine; TDF: tenofovir disoproxil; ABC: abacavir; FTC: emtricitabine; EFV: efavirenz; LPV: lopinavir; RPV: rilpivirine; RAL: raltegravir; BIC: bictegravir; EVG: elvitegravir; c: cobicistat; DOR: doravirine; HCV: hepatitis C virus; HBV: hepatitis B virus; TBC: tuberculosis; OBI: occult hepatitis B infection.

**Table 2:** Changes in immunologic and viremic observations during European Union stay (after 6–12 months).

Data	Szczecin, PL (n: 88)	Bonn, DE (n: 19)	*p* Value
Number of patients with CD4+ reconstruction/increase	45 (51.1%)	17 (62.9%)	*p* = 0.28
Mean CD4+ reconstruction (com/µL)	10 (IQR: −69.5–120.5)	51.5 (IQR: −22.5–135.5)	*p* = 0.36
Number of patients with undetectable viremia (<50 copies/mL)	78 (89.7%)	22 (95.7%)	*p* = 0.04

PL: Poland; DE: Germany.

## Data Availability

Data available upon request.
